# Association between formal thought disorder and cannabis use: a systematic review and meta-analysis

**DOI:** 10.1038/s41537-022-00286-0

**Published:** 2022-09-29

**Authors:** Mathilde Argote, Guillaume Sescousse, Jérôme Brunelin, Eric Fakra, Mikail Nourredine, Benjamin Rolland

**Affiliations:** 1 PSYR2, CNRL, INSERM U1028, CNRS UMR5292, UCBL1 Bron, France; 2 Université Claude Bernard Lyon 1, Lyon, France; 3 Centre Hospitalier Le Vinatier, Bron, France; 4 Pôle Universitaire de Psychiatrie, CHU Saint-Etienne, Saint-Etienne, France; 5 Service de biostatistique, Hospices Civils de Lyon, Lyon, France; 6 Service hospitalo-universitaire de pharmacotoxicologie, Hospices Civils de Lyon, Lyon, France; 7 Service Universitaire d’Addictologie de Lyon (SUAL), HCL, CH Le Vinatier, Lyon, France

**Keywords:** Schizophrenia, Psychosis, Schizophrenia

## Abstract

Formal thought disorder (FTD) is a multidimensional syndrome mainly occurring along the psychosis continuum. Cannabis use is known to increase symptoms of psychosis, particularly positive symptoms. However, the impact of cannabis use on FTD in individuals presenting symptoms along the psychosis continuum remains unclear. To address this knowledge gap, we conducted a meta-analysis examining the association between cannabis use and FTD in those individuals. We hypothesized that cannabis would worsen FTD. We conducted a systematic search of the PubMed, ScienceDirect, PsycINFO, Web of Science, Embase and Google Scholar databases up to July 2022. The results were collated through a random-effects model using the statistical software R. Reference lists of included studies were searched for additional relevant publications. Nineteen studies were included, totalling 1840 cannabis users and 3351 non-cannabis users. The severity of FTD was found to be higher in cannabis users (SMD = 0.21, 95%CI [0.12–0.29], *p* = 0.00009). Subgroup analyses revealed that FTD severity was increased among cannabis users, regardless of the disorder severity: healthy individuals (SMD = 0.19, 95%CI [0.05–0.33], *p* = 0.02); patients with first-episode psychosis (SMD = 0.21, 95%CI [0.01–0.41], *p* = 0.04); patients with schizophrenia (SMD = 0.25, 95%CI [0.11–0.38], *p* = 0.005). Between-group differences were not significant. In line with its already known effect on positive symptoms in psychosis, cannabis use appears to be associated with increased FTD severity all along the psychosis continuum. Future research should consider potential confounding variables such as other substance use disorders and explore how FTD dimensions are impacted by cannabis use.

## Introduction

Formal thought disorder (FTD) refers to the disruption of the process of thoughts and language production, clinically resulting in an alteration of effective communication^1,2^ and is regarded as a multidimensional syndrome^3^. FTD prevalence and severity are proportionally higher in patients with psychotic disorders than in healthy populations^2^. 6% of people in the general population and up to 80% of patients with psychotic disorders exhibit more or less pronounced symptoms of FTD, suggesting that FTD is best conceptualized as a continuous dimension^2^. FTD is particularly frequent in schizophrenia, as it is found in 27–80% of patients^1^, but it may also occur in other types of disorders such as depression and mania^1,4^. FTD encompasses particularly disabling symptoms that increase social isolation^5^, reduce quality of life^6^ and are associated with poorer clinical outcomes^7^. This disorder is predictive of conversion to psychosis in high-risk populations^8^, and is the strongest predictor of conversion from first-episode psychosis to schizophrenia^9^.

Cannabis use is one of the most prominent risk factors associated with the occurrence and severity of many psychotic disorders, including schizophrenia. In patients with schizophrenia, lifetime cannabis use is estimated at 42.1^10^ and 26.2% of patients with schizophrenia suffer from cannabis use disorder^11^. Cannabis use doubles the risk of developing a psychotic disorder in vulnerable individuals^12^, and triggers the onset of first-episode psychosis^13^ and psychotic disorders two to three years earlier than in non-users^14^. Among patients with schizophrenia, cannabis users present more severe positive symptoms, such as hallucinations and delusions^15^, whereas negative symptoms such as avolition and anhedonia seem less impacted by cannabis use^16^. FTD has a special place in the semiology of schizophrenia spectrum disorders, as it has been suggested to constitute a core (psychopathological) dimension of the disorder^17^. FTD dimensional structure is currently debated in the literature, leading to a high heterogeneity of assessment scales and conceptual definitions of this syndrome. At least two dimensions emerge as a consensus: *negative FTD*, referring to a deficit in speech and thought production; and *positive FTD*, referring to an increased amount of produced speech. Positive FTD is consistently associated with the disorganization dimension in several models of schizophrenia symptoms^1,7,18^. Previous research in individuals with schizotypal traits reported that the severity of disorganization was higher in cannabis users than in non-users^19^, suggesting that cannabis worsens positive FTD in a non-clinical sample.

The current review and meta-analysis firstly aimed to determine whether FTD and cannabis use are associated at different stages of the psychosis continuum, i.e. no or attenuated symptoms (stages 0, 1a, 1b), first-episode psychosis (stage 2), and recurrence and treatment resistance of the disorder (stages 3, 4)^20^. Subsequently, it aimed to investigate whether cannabis use would have a distinct impact on FTD depending on the stage of the psychotic disorder. We expected to find a positive association between cannabis use and FTD severity, all along the psychosis continuum.

## Methods

The reporting of this meta-analysis was guided by the standards of the updated Preferred Reporting Items for Systematic Reviews and Meta-Analyses guidelines (PRISMA)^21^. No pre-registration was performed for this meta-analysis.

### Search strategy

PubMed, ScienceDirect, Web of Science, PsycINFO, Embase and Google Scholar were used as sources to retrieve publications. The search syntax was as follows: [“thought disorder” OR “thought disturbance” OR “cognitive disorganization” OR “formal thought disorder” OR “disorganized speech” AND “cannabis” OR “marijuana” OR “THC” OR “pot” OR “hashish” OR “bhang” OR “ganja”] (see supplementary materials “SearchSyntax” for details). A filter was applied on ScienceDirect to only retrieve “research articles”. In addition, only the first 200 relevant sources were retrieved from Google Scholar, as recommended^22^. Each database supports spelling variations, such as the terms “disorganization” and “disorganized” were automatically searched with British and American spelling. Each source was last searched in July 2022. Reference lists of the selected publications were reviewed to identify additional relevant studies.

**Fig. 1:**
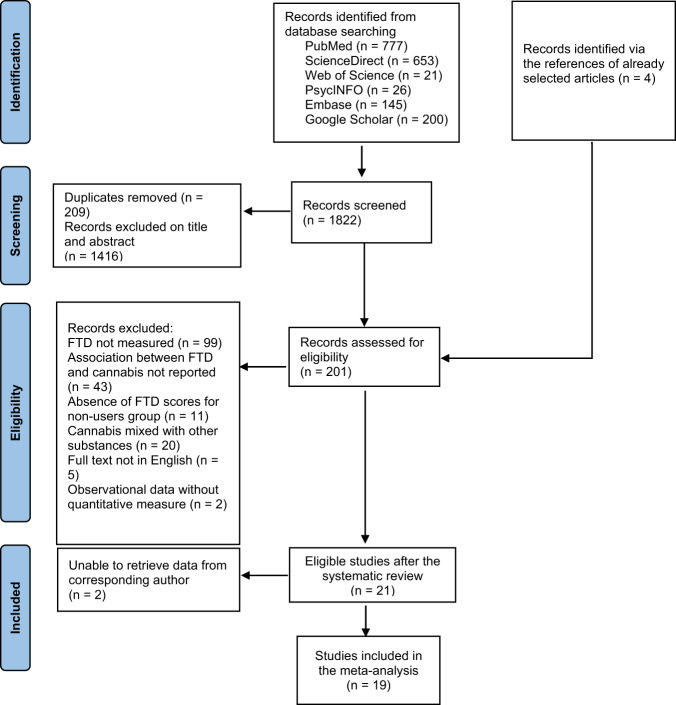
Prisma flow diagram describing the selection process of included publications.

Duplicates were removed by manual screening. After duplicate removal, each record was first screened on title and abstract by two independent researchers (MA and GS). In case of disagreement, the inclusion of the study was decided by a third researcher (BR). A second selection round was based on full text reading and followed the same procedure. The flow-chart detailing the selection process is provided in Fig. 1.

### Eligibility criteria

To be included, each study had to meet the following criteria: (1) report FTD scores of two distinct groups: one of cannabis users and a control one of non-users; (2) include individuals presenting symptoms pertaining to the psychosis continuum; (3) use validated tools for assessing FTD; and (4) be written in English. Studies were excluded if: (1) FTD was not specifically distinguished from other psychotic symptoms, (2) cannabis use was not assessed, or not distinguished from the use of other substances, (3) the link between FTD and cannabis use was not reported in the analyses.

### Data extraction

Data used for statistical analyses were extracted from the Results section or the supplementary material part of selected studies. To allow for a comparison between groups, data had to be extracted into two groups: one group of participants using cannabis and one of participants not using cannabis. In addition, both extracted groups of participants had to present symptoms pertaining to the same stage of the psychosis continuum (i.e. patients with schizophrenia and not using cannabis compared to patients with schizophrenia using cannabis). In cases where study designs included participants pertaining to multiple groups in terms of their drug use (i.e. polysubstance users, cannabis mainly users, alcohol mainly users, non-users), only the relevant groups were extracted (i.e. cannabis mainly users and non-users). Similarly, in the event that multiple groups of participants were formed in terms of the disorder severity (i.e. first-episode psychosis compared to healthy controls), only data of the relevant group was extracted (i.e. first-episode psychosis using and not using cannabis).

In cases where insufficient data was present for the calculation of our primary effect size, the principal investigators were contacted via email^13,23–25^. Two of the contacted authors provided the requested data^13,23^.

### Quality assessment

The risk of bias was assessed by two independent researchers (MA and MR) using the Quality Assessment Tool for Observational Cohort and Cross-sectional studies^26^, a recommended tool for analytical cross-sectional studies^27^. This process ensures that the quality of included studies is good enough to provide reliable results. Based on a series of questions, the goal is to identify potential flaws in the publication that may impact the outcome measure we want to study. Quality of studies can be rated as “poor”, “fair”, or “good”. Question 9 “Were the exposure measures (independent variables) clearly defined, valid, reliable, and implemented consistently across all study participants?” and question 11 “Were the outcome measures (dependent variables) clearly defined, valid, reliable, and implemented consistently across all study participants?” were judged crucial as the focus for the current meta-analysis is the relationship between FTD (outcome) and cannabis use (exposure). Question 14 “Were key potential confounding variables measured and adjusted statistically for their impact on the relationship between exposure(s) and outcome(s)?” was considered important as the presence of confounding variables can distort the relationship between FTD and cannabis use. In cases where studies collected a “no” to questions 9 and 11, the quality was not rated as “good”.

### Meta-analysis methods

Analyses were conducted using the statistical software R version 4.1.1 (R Core Team 2021)^28^. Mean scores, standard deviations and binary outcomes (FTD present or absent) of each study were extracted for cannabis users and for non-users; they were subsequently converted into between-groups Standardized Mean Differences (SMD) using the {esc} package, so that the results obtained from different scales could be pooled together. In cases where studies reported the result of a two-sample t-test^29^, the t-statistic was converted into a SMD. Hedge’s g correction was applied to all SMDs to correct for small-sample size. In cases where studies categorized groups into types of cannabis used (i.e. skunk, herbal, mixed)^30^, or level of use (i.e. use, harmful use, Substance Use Disorder)^13^, we regrouped these cannabis users into a single group for each study by calculating their mean scores and standard deviations. SMDs were calculated comparing the statistics of the cannabis-using groups with those of the non-using groups. In cases where SMD values are positive, cannabis-using groups present higher FTD mean scores than non-using groups. When SMD values are negative, cannabis-using groups present lower FTD mean scores than non-using groups. The overall calculated effect size was then transformed back into natural units of two widely used scales (Positive and Negative Symptoms Scale and Scale for the Assessment of Positive Symptoms) to facilitate interpretation of the results. This transformation is based on a recognized statistical method^31–33^; it was obtained by multiplying the calculated SMD by the pooled standard deviation of selected scales^31,34^. The interpretation values for effect size (SMD) according to Cohen’s classification are: 0.1–0.3 (small effect), 0.3–0.5 (moderate effect) and superior to 0.5 (large effect).

The meta-analysis was performed using the {meta} package. Group combination, plot generation and outlier identification were performed using the {dmetar} package. Between-study heterogeneity was computed via a random effect model with I², where I² = 25%, I² = 50%, and I² = 75% are considered as low, moderate, and high heterogeneity, respectively^35^. Tau² was estimated using the Restricted Maximum Likelihood. In addition, Hartung-Knapp adjustment^36^ was applied to the 95% confidence interval of the pooled effect size to reduce the chances of false positives. The prediction interval was calculated to estimate the reliability of the results in the context of future studies. The search for outliers was performed to address between-study heterogeneity, using the “find.outliers” function included in the {dmetar} package. A sensitivity analysis was conducted to exclude cases that would influence too greatly the results, due to a large sample size. This method allows to visualize if the calculated SMD was distorted by one large study influencing the true effect size. This supplementary analysis ensures that the synthetized results were not distorted and thus robust. In cases where the influence analysis detected studies affecting too greatly the results, the statistical leave-one-out method was applied to confirm those cases. This method estimates the overall effect size omitting one study at a time. In addition, we performed post hoc subgroup analyses to investigate the effect of cannabis use on the different populations included across publications (i.e. no or attenuated symptoms, first-episode psychosis, schizophrenia). Publication bias was visually inspected with a funnel plot and quantified with an Egger’s test measuring the asymmetry of the funnel plot.

## Results

The flow diagram of the study selection process is provided in Fig. 1. Searches yielded 1822 results, which were then refined by the screening of titles and abstracts. After the screening process, 201 publications were assessed for eligibility based on a full-text reading, leading to the selection of 21 relevant studies. Details regarding the reasons for study exclusion are provided in Fig. 1. We finally included 19 publications in the meta-analysis because data extraction could not be performed for two studies^24,25^.

Across the 19 studies, a total of 5191 participants were included: 1840 in the cannabis-users group and 3351 in the non-users group.

### Effect measures

In the various studies, FTD was referred to as “cognitive disorganization”^30,37–39^, “disorganization“^23,40–45^, “thought disturbance”^29^, “FTD”^46,47^ or “positive FTD”^13,48–51^. This is linked to the scale used (see supplementary material “Scales” for details). Each of these terms refers to a similar concept of FTD, encompassing speech abnormalities, poor attention, conceptual disorganization, and difficulties in thought process. For clarity, the umbrella term “FTD” will be used to refer to all these terms throughout this meta-analysis. Eight studies^23,30,37–39,42,44,45^ assessed FTD globally using two questionnaires, the Oxford-Liverpool Inventory of Feelings and Experiences^52^ and the Schizotypal Personality Questionnaire^53^, as well as three scales: the Positive and Negative Syndrome Scale^54^, the Psychotomimetic States Inventory^52^, and the Structured Interview for Prodromal Symptoms^45^. Nine studies^13,29,40,41,46,48–51^ evaluated subcomponents of FTD using the Scale for the Assessment of Positive Symptoms^55^, the Brief Psychiatric Rating Scale^56^, the Young Mania Rating Scale^57^, the Operational Criteria for Psychotic Illness^58^ and the Symptom Onset in Schizophrenia^59^. The main subcomponent assessed via these scales was positive FTD, characterized by disorganized speech. One study reported results for both negative and positive FTD^48^. Since the other studies focused on either the global concept of FTD^13,29,30,37,47^, which includes positive FTD, or only positive FTD^23,46,50,51^, this dimension was the only one considered in order to maintain homogeneity between compared outcomes. Some studies also reported results as a proportion of participants in whom FTD was either present or absent^40,47,48,51^.

### Study characteristics

The eligible studies included different types of population: (1) individuals with no history of diagnosed mental illness^23,30,38,39,42–45^; (2) individuals with a history of first-episode psychosis^13,37,40,46^; (3) individuals with schizophrenia^29,41,48–51^, according to validated diagnosis tools; and (4) individuals with cannabis-induced psychosis^47^, as determined by an expert. For the purpose of this meta-analysis, it was decided to form four distinct groups of individuals, according to the staging model of psychotic disorders^20^: (1) individuals without symptoms, or attenuated syndrome (stages 0, 1a, 1b); (2) individuals with first-episode psychosis (stage 2); (3) individuals with schizophrenia (stages 3–4); and (4) individuals with cannabis-induced psychosis (Table 1).

**Table 1:** Studies included in review and meta-analysis.

Study reference - country	Design	Extracted population^a^	Symptom (assessment mode)	Key findings
Basu 1999^47^ - India	Retrospective	24 CIP Patients 20 acute schizophrenic episode without history of use	FTD (from case reports)	Percentage of FTD per group: CIP group: 15% Acute schizophrenic episode group: 60%
Boydell 2007^48^ - UK	Retrospective	182 SCZ + CU 552 SCZ nCU	Positive FTD (OPCRIT checklist from case reports)	Percentage of pFTD per group: CU group: 32% nCU group: 25%
Caspari 1999^29^ - Germany	Longitudinal	39 SCZ + CUD 39 SCZ nCU	Thought disturbance (BPRS)	Thought disturbance: t-test between groups: t = 2.25
Cohen 2011^23^ - USA	Cross-sectional	20 Schizotypy + CU 74 Schizotypy nCU	Disorganization (SPQ-BR)	Disorganization mean (sd) scores: CU group: 31.31 (3.36) nCU group: 30.58 (4.33)
Dubertret 2006^51^ - France	Cross-sectional	38 SCZ + CUD 121 SCZ nCU	Positive FTD (SAPS)	Percentage of pFTD per group: CUD group: 84% nCU group: 85%
Gonzales-Blanco^40^ - Spain	Retrospective	144 FEP + CU 70 FEP nCU	Disorganization (SOS)	Percentage of Disorganization per group: CU group: 63.9% nCU group: 58%
Herzig 2015^37^ - Switzerland	Cross-sectional	11 FEP + CU 18 FEP nCU	Cognitive Disorganization (PANSS)	CogDis mean (sd) scores: CU group: 6.10 (3.36) nCU group: 4.45 (1.86)
Ho 2011^41^ - USA	Cross-sectional	52 SCZ + CUD 183 SCZ nCU	Disorganization (SAPS subscore)	Disorganization mean (sd) scores: CU: 5.1 (3.0) nCU: 4.8 (3.0)
Koen 2009^49^ - South Africa	Retrospective	245 SCZ + CU/CUD 302 SCZ nCU	Positive FTD (SAPS)	pFTD mean (sd) scores: CU: 1.46 (1.58) nCU: 1.02 (1.38)
Korver 2010^45^ - The Netherlands	Cross-sectional	34 CU 29 nCU	Disorganization (SIPS)	Disorganization mean (sd) scores: CU: 4.59 (2.3) nCU: 5.17 (3.21)
Mackie 2021^30^ - UK	Cross-sectional	143 CU 323 nCU	Cognitive disorganization (O-LIFE)	CogDis mean (sd) scores: nCU:5.26 (2.7) CU: 5.35 (3.03)
Mason 2008^39^ – UK	Repeated measures	140 CU 144 nCU	Cognitive Disorganization (PSI)	CogDis mean (sd) scores: CU: 3.7 (3.4) nCU: 3.5 (4.0)
Nunn 2001^38^ – UK	Cross-sectional	49 CU 49 no drinking/drug-using	Cognitive disorganization (O-LIFE)	CogDis mean (sd) scores: CU: 13.20 (2.33) no drink/drug: 12.76 (1.26)
O’Tuathaigh 2020^42^ - Ireland	Cross-sectional	181 CU 563 nCU	Disorganization (SPQ)	Disorganization mean (sd) scores: CU: 6.67 (4.03) nCU: 5.29 (4.11)
Peralta 1992^50^ - Spain	Cross-sectional	23 SCZ + CUD 72 SCZ nCU	Positive FTD (SAPS)	pFTD mean (sd) scores: CUD: 2.9 (1.8) nCU: 2.2 (1.7)
Pope 2021^46^ - USA	Cross-sectional	155 FEP + CU 76 FEP nCU	Positive FTD (SAPS)	pFTD mean (sd) scores: nCU: 6.4 (6.2) CU: 6.9 (6.3)
Schiffman 2005^43^ - USA	Cross-sectional	43 CU 146 nCU	Disorganization (SPQ-B)	Disorganization mean (sd) scores: CU: 2.40 (1.78) nCU: 1.71 (1.71)
Soler 2018^44^ - Spain	Cross-sectional	110 CU 275 nCU	Disorganization (SPQ)	Disorganization mean (sd) scores: CU: 1.4 (1.31) nCU: 1.06 (1.20)
Stone 2014^13^ - UK	Longitudinal	207 FEP + CU 295 FEP nCU	Thought Disorder (YMRS)	TD mean (sd) scores: CU: 0.77 (1.04) nCU: 0.52 (0.9)

Abbreviations: *CIP* Cannabis Induced Psychosis, *CUD* Cannabis Use Disorder, *CU* Cannabis Users, *nCU* non-Cannabis Users, *AL* Alcohol-using, *SCZ* Schizophrenia, *FEP* First Episode Psychosis, *SUD* Substance Use Disorder; *FTD* Formal Thought Disorder, *pFTD* Positive Formal Thought Disorder, *nFTD* Negative Formal Thought Disorder, *TD* Thought Disorder, *sd* Standard Deviation.

^a^Refers to the population of interest extracted from original publications.

### Risk of bias

The overall risk of bias was considered as low: the quality of four studies was rated as “good”^29,37,44,51^, that of fourteen studies as “fair”^13,23,30,38,40–43,45,46,48–50,52^ and that of one as “poor”^47^. Poor quality was firstly determined by the imprecise criteria for pertaining to the cannabis using group, i.e. “regular cannabis use for at least 1 month prior to onset of psychosis”, and by the imprecise measure of FTD in participants, i.e. “no psychopathology scale could be used”^47^. Most studies were rated as “fair” quality as either question 9 or question 11 of the Quality Assessment Tool for Observational Cohort and Cross-sectional studies rated as “yes” for these publications. Good quality was decided when the questions 9, 11, and 14 rated as “yes”, as well as the other questions of the tool. The detailed ratings can be found in the supplementary material.

### Cannabis Use and FTD

**Fig. 2:**
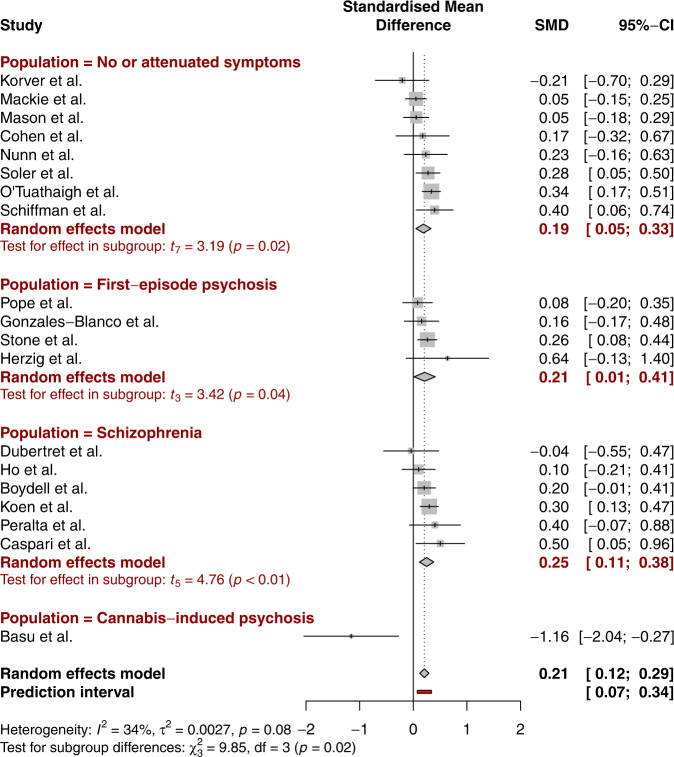
Forest plot displaying results of original analysis combining individual studies and subgroup analyses, standardized mean difference (SMD), its confidence interval (CI), and overall prediction interval (PI), plus the weight of each study.

The overall effect size of the 19 pooled studies indicated that FTD severity was significantly increased in cannabis-users groups, compared to non-using groups (SMD = 0.21, *p* = 0.00009, 95% CI [0.12; 0.29]). Between-group heterogeneity was moderate (I² = 34%, *p* = 0.08). The estimated range of the prediction interval indicates that the effect size may lie between 0.07 and 0.34 in the context of future studies on the same issue. Original results are presented in Fig. 2.

### Sensitivity analyses

The study rated as being of “poor” quality was reported as an outlier^47^. While its inclusion in the original analysis had a negligible impact on the overall results, it greatly affected heterogeneity (Table 2. “Main” analysis for statistical details). Considering its outlier status and its large impact on heterogeneity, it was excluded from the other analyses performed. Thus, the overall effect size of the 18 remaining studies was kept throughout the current meta-analysis (SMD = 0.22, *p* = 0.000006, 95% CI [0.14; 0.53]), with a low between-study heterogeneity (I² = 4.8%, *p* = 0.4). Prediction interval based on the 18 remaining studies confirmed that the effect size may lie within a range of positive values (PI [0.10; 0.33]). A second sensitivity analysis was performed, excluding two studies considered as having a large influence on the results by the statistical leave-one-out method^30,42^ (see supplementary material “InfluenceAnalysis”). The removal of these two studies did not significantly impact the overall effect size, which means that their influence did not distort the calculated effect size in the first analysis (SMD = 0.22, *p* < 0.0001, 95% CI [0.15; 0.29], I² < 1%, *p* = 0.59). Table 2 provides a detailed explanation of the statistical results of the two distinct sensitivity analyses performed.

**Table 2:** Results from the two sensitivity analyses conducted: first removing the outlier; then the high-influence studies from the original analysis.

Analysis	SMD	95%CI	*p*	95%PI	I²	I² 95%CI
Original^a^	0.21	[0.12;0.29]	0.00009	[0.10;0.33]	34%	[0.0%;61.9%]
Main - Outlier removed^b^	0.22	[0.15;0.29]	<0.0001	[0.10;0.33]	5%	[0.0%;52.4%]
Infl. studies removed^c^	0.22	[0.15;0.29]	<0.0001	[0.14;0.30]	<1%	[0.0%;52.3%]

^a^All studies included (*k* = 19). ^b^Outlier excluded^47^(*k* = 18). ^c^Three studies excluded^30,42,47^ (*k* = 16). Abbreviations: *k*, number of studies, *CI* Confidence Interval; *PI* Predication Interval, *Infl. studies* Influence studies.

When the SMD was back transformed using the pooled standard deviation of the PANSS, the mean difference in Cognitive Disorganization score between cannabis-using and non-using groups equals to 0.57 points; Cognitive Disorganization scores ranged from 3 to 21 points. Applied to the SAPS, the mean difference in positive FTD score between cannabis-using and non-using groups equals to 0.93 points; positive FTD ranged from 0 to 45 points.

### Subgroup analyses

Subgroup analyses were performed to examine the effect of cannabis use on the groups formed in line with the staging model of psychotic disorders previously mentioned: (1) individuals with no or attenuated symptoms, (2) patients with first-episode psychosis, and (3) patients diagnosed with schizophrenia. The cannabis-induced psychosis group was not represented as the only study focusing on this population was the outlier and thus excluded. Individuals with no or attenuated symptoms were extracted from the general population and are thus considered a prodromal population. First-episode psychosis patients were described in the publications as having experienced psychotic symptoms accompanied by a decrease in functioning over the month before inclusion^37^, being diagnosed with a primary non-affective psychotic disorder^46^, having a history of psychotic symptoms that lasted for more than 7 days^13^, experiencing the first episode of schizophrenia, excluding brief psychotic episodes due to intoxication^48^, or experiencing a first occurrence of positive and negative, cognitive and affective symptoms^40^. Individuals using cannabis in the first group (no or attenuated symptoms) demonstrated significantly higher FTD scores compared to non-users (SMD = 0.19, 95% CI [0.05;0.33], *p* = 0.02). Cannabis users in the second group (first-episode psychosis) also had a higher FTD severity than non-users, the 2 groups showed a comparable effect size (SMD = 0.21, 95% CI [0.01;0.41], *p* = 0.04). Although tests revealed that the differences in effect size were not significant between groups (*p* = 0.75), cannabis users with a diagnosis of schizophrenia displayed higher FTD severity compared with non-users, with the highest effect size (SMD = 0.25, 95% CI [0.11;0.38], *p* = 0.005).

### Publication bias

**Fig. 3:**
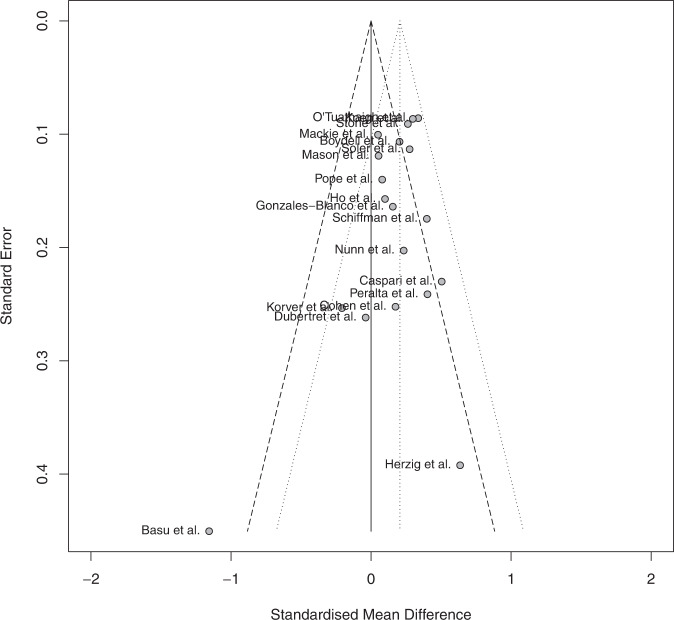
Funnel plot investigating publication bias.

Studies reporting low effect sizes may remain unpublished because of non-significant results. Only studies reporting significant results tend to be published, creating a publication bias. This bias can be visually and statistically assessed via the inspection of a funnel plot and the statistical Egger’s test. The visual inspection of the funnel plot indicates a relatively low asymmetry. However, one study appears far away from the ideal delineated area (see Fig. 3). It is the same study that was found to be an outlier and excluded from the analysis^47^. Egger’s test was performed on the nineteen studies to statistically assess the publication bias (supplementary material “EggerTest”). The Egger’s test did not reveal significant presence of asymmetry, indicating no evidence for a publication bias.

## Discussion

This meta-analysis aimed to review the relationship between cannabis use and FTD along a continuum ranging from individuals with no history of characterized mental disorders to patients meeting the criteria for schizophrenia, what had never been performed to our knowledge. The main results of the global analysis showed that individuals using cannabis presented a significantly higher severity of FTD than non-users. Although the effect was significant, the size of the effect (SMD = 0.20) should be considered as small^60^. Subgroup analyses were then performed to investigate whether the relationship between cannabis use and FTD varied between individuals of the included populations: no or attenuated symptoms, first-episode psychosis, and schizophrenia. Cannabis users from all three subgroups exhibited a similar FTD severity, suggesting a stable association independent of the underlying stage of the psychotic disorder. Thus, the association between FTD and cannabis use should not be seen as an intermediate vulnerability marker for schizophrenia. However, since both cannabis use and FTD severity were associated with increased risk for psychosis, we cannot rule out a role of the relationship between FTD and cannabis use in the psychotic transition. Indeed, triggering factors of psychotic symptoms are complex and combine both biological features such as genetic traits, and environmental factors such as socio-economic or migration status^61^. The results support the hypothesis that cannabis use could be seen as one of the key factors explaining the severity of FTD.

The results are in line with previous findings where disorganization symptoms were exacerbated by cannabis use in individuals with schizotypal traits^19^. In addition, some studies described an aggravation of disorganization when individuals at a prodromal stage of psychosis present an acute cannabis intoxication^62,63^. Findings from a ten-year longitudinal experiment, which investigated the impact of cannabis use on the course of illness in schizophrenia, reported that compared to non-users, cannabis users presented higher scores of disorganized symptoms at the 6-month, 4-year and 10-year follow-ups^64^, implying an aggravation of FTD by cannabis use, in line with the findings presented in the current review. In the same study, however, a decrease in disorganized symptoms for some individuals was associated with a higher likelihood of cannabis use^64^. This could mean that some individuals use cannabis to alleviate their disorganized symptoms^64^, demonstrating the need to distinguish between subjective and objective FTD^3,65^.

This meta-analysis has some strengths. Publication bias, as well as the risk of bias in each individual study, and small-sample study biases, were taken into account in our meta-analysis. Between-study heterogeneity is considered as very low, due to the use of a random model and the outlier identification. All the relevant methods that avoid bias were employed to provide the most reliable results. However, there are several factors that could not be controlled for as this meta-analysis used aggregated data. Because the included studies were epidemiological investigations, it is impossible to ascertain a causal effect of cannabis use on the severity of FTD; intermediary factors such as sociodemographic features, the use of cannabis for self-medication, the effect of antipsychotic treatment, or the presence of Substance Use Disorder (SUD) cannot be ruled out. SUDs involving cocaine, alcohol, stimulants, hallucinogens, or tobacco are associated with FTD severity^13,66,67^. Another identified limitation is that, even if the search syntax used in the current meta-analysis intended to include all publications on FTD and its subcomponents, FTD is actually a heterogeneous construct which can be expressed using many terms, and whose exact conceptual structure is still being explored using factor analyses of clinical scales^7^. Furthermore, there is a paucity of publications exploring the association between cannabis use and specific FTD subcomponents. Hence, FTD subcomponents other than the positive dimension could not be adequately represented in the current study. Previous works found a significant positive association between cannabis use and negative FTD^48^. This finding should be replicated, as it could broaden knowledge on this issue. In addition, subcomponents of FTD need to be more systematically explored in future research via the use of scales specifically designed to measure FTD severity such as the Thought And Language Disorder (TALD), the Thought Language and Communication Disorders (TLC), the Thought and Language Index (TLI), and the Thought Disorder Index (TDI)^1^. Finally, a last limitation of the current meta-analysis is to not have been preregistered in a public registry.

## Conclusion

Previous studies found that cannabis use was associated with more severe positive symptoms of psychosis, within an epidemiological continuum ranging from the general population to schizophrenia spectrum disorders. Moreover, accumulating pieces of evidence suggest a causal role of cannabis in triggering and aggravating positive symptoms, particularly in schizophrenia spectrum disorders. Since FTD has been suggested to constitute a core symptom of schizophrenia, it was theoretically expected that cannabis use would be associated with exacerbated FTD. The results presented in this meta-analysis tend to support this association, and the effect of cannabis use on FTD seems consistent along the psychosis continuum, regardless of the severity of the psychotic disorder. Intermediary factors need to be controlled for in future research on this issue. These findings encourage future research to assess FTD using specific scales and support the clinical relevance of assessing FTD in early stages of the psychosis continuum, specifically among individuals using cannabis.

## Supplementary information

Supplementary Material

## Data Availability

The data supporting the findings of this study are available within the paper and its supplementary information files.

## Code availability

The code written for the analyses will be available upon request.
