# Supplementary material for: Association between formal thought disorder and cannabis use: a systematic review and meta-analysis
Source: Schizophrenia (Heidelb). 2022 Sep 29;8(1):78. doi: 10.1038/s41537-022-00286-0 (PMC9523063; doi:10.1038/s41537-022-00286-0)
Supplement: Supplementary file 1 — Supplementary Material [file 41537_2022_286_MOESM1_ESM.docx]

**Table 1. Scales used in the studies: the concept assessed and its related symptoms**

| **Scale** | **Concept** | **Symptoms** |
| --- | --- | --- |
| O-LIFE | Cognitive Disorganization | Poor attention, concentration, decision-making |
| SPQ-B | Disorganization | Odd speech and behavior |
| PANSS | Cognitive Disorganization | Poor attention, difficulties in abstract thinking, conceptual disorganization |
| SAPS | Positive FTD | Speech abnormalities |
| BPRS | Thought disturbance | Conceptual disorganization, grandiosity, hallucinatory behavior, unusual thought content |
| YMRS | Language-thought disorder | Disrupted thoughts, speech abnormalities |
| OPCRIT | Positive FTD – negative FTD | Speech abnormalities - difficulties in concrete thinking |
| PSI | Cognitive Disorganization | Poor attention, flight of ideas |
| SOS | Disorganization | Disorganized thought process and behavior |
| SIPS | Disorganization | Odd behavior or appearance, bizarre thinking, trouble with focus and attention, personal hygiene |

Abbreviations: O-LIFE; Oxford-Liverpool Inventory of feelings and Experiences; SPQ-B, Schizotypal Personality Questionnaire-Brief; PANSS; Positive and Negative Symptoms Scale; SAPS, Scale for the Assessment of Positive Symptoms; BPRS, Brief Psychiatric Rating Scale; YMRS, Young Mania Rating Scale; OPCRIT, Operational Criteria Checklist for Psychotic Illness and Affective Illness; PSI, Psychotomimetic States Inventory; SOS, Symptom Onset in Schizophrenia; SIPS, Structured Interview for Prodromal Symptoms

**Table 2. Study quality assessment: ratings**

|  | Q1 | Q2 | Q3 | Q4 | Q5 | Q6 | Q7 | Q8 | Q9 | Q10 | Q11 | Q12 | Q13 | Q14 | Rate |
| --- | --- | --- | --- | --- | --- | --- | --- | --- | --- | --- | --- | --- | --- | --- | --- |
| Basu | Yes | Yes | C.D | Yes | No | N.A | Yes | N.A | No | N.A | No | No | N.A | No | Poor |
| Boydell | Yes | Yes | C.D | Yes | Yes | No | No | N.A | No | N.A | Yes | N.R | N.A | Yes | Fair |
| Caspari  Cohen | Yes  Yes | Yes  Yes | C.D  C.D | Yes  Yes | No  No | Yes  Yes | Yes  No | N.A  N.A | Yes  Yes | No  N.A | Yes  Yes | N.R  N.R | Yes  N.A | Yes  No | Good  Fair |
| Dubertret | Yes | Yes | C.D | Yes | Yes | No | No | N.A | Yes | N.A | Yes | N.R | N.A | Yes | Good |
| Gonzales-Blanco | Yes | Yes | C.D | Yes | No | N.A | No | N.R | Yes | N.A | Yes | N.R | N.A | No | Fair |
| Herzig | Yes | Yes | C.D | Yes | No | No | No | N.A | Yes | N.A | Yes | N.R | N.A | Yes | Good |
| Ho | Yes | Yes | C.D | Yes | No | No | No | No | Yes | N.A | Yes | N.R | N.A | No | Fair |
| Koen | Yes | Yes | C.D | Yes | No | No | No | N.A | Yes | N.A | Yes | N.R | N.A | No | Fair |
| Korver | Yes | Yes | C.D | Yes | No | No | No | No | No | N.A | Yes | N.R | N.A | Yes | Fair |
| Mackie | Yes | Yes | C.D | Yes | No | No | No | Yes | Yes | N.A | Yes | N.R | N.A | No | Fair |
| Mason | Yes | Yes | C.D | Yes | No | Yes | No | N.A | No | N.A | Yes | N.R | Yes | Yes | Fair |
| Nunn | Yes | Yes | C.D | Yes | No | No | No | N.A | No | N.A | Yes | N.R | N.A | Yes | Fair |
| O’tuathaigh | Yes | Yes | C.D | Yes | No | No | No | Yes | Yes | N.A | Yes | N.R | N.A | No | Fair |
| Peralta | Yes | Yes | C.D | Yes | No | No | No | N.A | Yes | N.A | Yes | N.R | N.A | No | Fair |
| Pope | Yes | Yes | C.D | Yes | No | No | No | N.A | Yes | N.A | Yes | N.R | N.A | No | Fair |
| Schiffman | Yes | No | C.D | Yes | No | No | No | N.A | Yes | N.A | Yes | N.R | N.A | No | Fair |
| Soler | Yes | Yes | C.D | Yes | No | No | No | Yes | Yes | N.A | Yes | N.R | N.A | Yes | Good |
| Stone | Yes | Yes | C.D | Yes | No | Yes | Yes | Yes | Yes | Yes | Yes | N.R | No | No | Fair |

Abbreviations: N.A, Not Applicable; CD, cannot determine; N.R, Not Reported

**Search syntax:**

**PubMed**

((thought disorder) OR (formal thought disorder) OR (thought disturbance) OR (cognitive disorganization) OR (disorganized speech)) AND ((cannabis) OR (THC) OR (marijuana) OR (pot) OR (hashish) OR (bhang) OR (ganja))

**ScienceDirect***

("thought disorder" OR "formal thought disorder" OR "thought disturbance" OR "cognitive disorganization" OR "disorganized speech") AND ("cannabis" OR "THC" OR "marijuana" OR “pot” OR “hashish” OR “bhang” OR “ganja”))

*Filtered for research articles only.

**Web of Science**

((“thought disorder” OR “thought disturbance” OR “cognitive disorganization” OR “formal thought disorder” OR “disorganized speech”) AND (“cannabis” OR "THC" OR "marijuana" OR "pot" OR "hashish" OR "bhang" OR "ganja"))

**PsycINFO and Embase**

(“thought disorder” OR “thought disturbance” OR “cognitive disorganization” OR “formal thought disorder” OR “disorganized speech”) AND (“cannabis” OR "THC" OR "marijuana" OR “pot” OR “hashish” OR “bhang” OR “ganja”)

**Google Scholar***

(“thought disorder” OR “thought disturbance” OR “cognitive disorganization” OR “formal thought disorder” OR “disorganized speech”) (“cannabis” OR "THC" OR "marijuana" OR “pot” OR “hashish” OR “bhang” OR “ganja”)

*First 200 relevant sources retrieved

**Last search:**

08/07/2022

| Intercept | 95%CI | t | p |
| --- | --- | --- | --- |
| -0.395 | -2.15 - 1.36 | -0.442 | 0.67 |

**Table 3. Results of Egger’s Test**

Eggers' test does not indicate the presence of funnel plot asymmetry


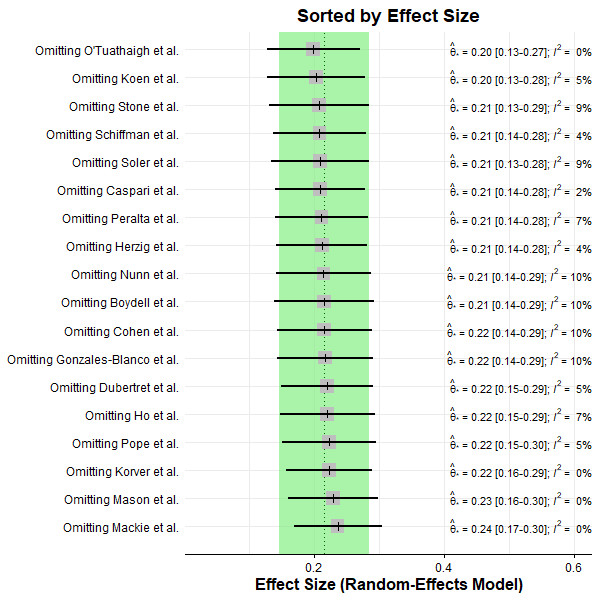


**Supplementary figure. Influence analysis**
